# Effect of rapid maxillary expansion on masticatory and swallowing functions in children with posterior crossbite^☆^

**DOI:** 10.1016/j.bjorl.2023.101304

**Published:** 2023-08-19

**Authors:** Tais H. Grechi, Carla E. Itikawa, Fernanda W.M. Gallarreta, Wilma T. Anselmo-Lima, Fabiana C.P. Valera, Luciana V.V. Trawitzki

**Affiliations:** a Universidade de São Paulo, Faculdade de Medicina de Ribeirão Preto, Hospital das Clínicas, Ribeirão Preto, SP, Brazil; b Universidade de São Paulo, Faculdade de Odontologia de Ribeirão Preto, Ribeirão Preto, SP, Brazil; c Universidade de São Paulo, Faculdade de Medicina de Ribeirão Preto, Departamento de Oftalmologia, Otorrinolaringologia e Cirurgia de Cabeça e Pescoço, Ribeirão Preto, SP, Brazil

**Keywords:** Crossbite, Rapid maxillary expansion, Oral physiology

## Abstract

**Objective:**

Investigate the influence of posterior crossbite on masticatory and swallowing functions by videofluoroscopy examination and to analyze the effects of Rapid Maxillary Expansion (RME) on the same functions.

**Methods:**

A prospective longitudinal study was conducted on 32 children, 21 of them with posterior crossbite (10 girls and 11 boys, mean age 9.2 years, study group) and 11 children with no occlusal changes (6 girls and 5 boys, mean age 9.3 years, control group). The children were evaluated by an orthodontist for diagnosis, group characterization and occlusal treatment, by the otorhinolaryngology team for the assessment of respiratory symptoms, and by a speech therapist for videofluoroscopic evaluation of masticatory and swallowing parameters. The children with posterior crossbite were treated orthodontic ally using the Haas disjunctor for approximately six months and re-evaluated 5-months after removal of the appliance. Data were analyzed statistically by the Student*t*-test for independent samples for comparison of the crossbite and control groups before and after treatment.

**Results:**

There was no significant difference in oral preparatory and oral transit times, nor in cycles and masticatory frequency, between the pre and post orthodontic treatment groups and the control group.

**Conclusion:**

Rapid maxillary expansion did not influence the masticatory and swallowing variables studied by videofluoroscopy. However, the data require caution in interpretation.

## Highlights

• Multidisciplinary study pre and post orthodontic intervention.
• Study by using of use videofluoroscopy before and after orthodontic treatment.
• Rapid maxillary expansion did not influence the studied masticatory and swallowing variables.

## Introduction

Posterior crossbite is defined as malocclusion in the canine, premolar and molar regions, with the vestibular cusps of the upper teeth lingually occluding the vestibular cusps of the corresponding lower teeth.^1^ It can be unilateral or bilateral and may be present in the different phases of dentition.

Several studies have related posterior crossbite to the presence of deleterious oral habits, orofacial myofunctional disorders and mouth breathing.^2^, ^3^, ^4^ Posterior crossbites one of the most frequent types of malocclusion during the phase of deciduous and mixed dentition, with a prevalence of 7.2%–23%.^5^ It may cause changes in mandibular symmetry,^6^ in masticatory coordination and pattern,^7^, ^8^ in swallowing pattern,^4^ and in bite strength.^8^

One of the orthodontic treatments frequently used for the correction of posterior crossbites Rapid Maxillary Expansion (RME) in order to increase the perimeter of the upper arch by opening the midpalatal suture.^9^, ^10^ The effects of RME on the orofacial myofunctional condition are still unclear and few longitudinal studies investigating these aspects are available.

Viedofluoroscopy is an instrument that permits a dynamic and detailed analysis of orofacial functions, with real time recording of the oral, phryngeal and laryngeal events.^11^ The exam is used in clinical speech therapy practice as an auxiliary method for the diagnosis of swallowing disorders.^12^

The hypothesis of the present study was that posterior crossbite and its orthodontic correction by RME may induce changes in masticatory and swallowing patterns as determined by videofluoroscopy.

## Methods

The evidence level of the present study is level 4 (Oxford Center for Evidence-Based Medicine 2011).^13^

### Subject selection

Thirty-nine children, 20 boys and 19 girls aged on average 9.3 years, in the phase of mixed dentition, initially participated in the study. The children were divided into two groups, 28 subjects with posterior crossbite (posterior crossbite group) and 11 with no occlusal changes (control group). The children were selected at the Screening Office of the Preventive and Interceptor Orthodontic Clinic of the Dental School and at the Mouth Breather Center (CERB) and at the Otorhinolaryngology service of the University Hospital, School of Medicine of Ribeirão Preto, University of São Paulo. The study was approved by the Research Ethics Committee (protocol number 6443/2007) and all persons responsible for the children gave written informed consent to participate.

Inclusion criterion for the crossbite group was the presence of unilateral or bilateral crossbite involving the deciduous canines and molars and the first permanent molars, with an indication of orthodontic treatment, with no restrictions regarding respiratory condition or other associated malocclusions. The inclusion criteria for the control group were absence of occlusal changes, being in the mixed dentition phase, no history of orthodontic and/or orthopedic treatment, and no restrictions regarding respiratory condition.

Exclusion criteria were presence of genetic syndromes, congenital and acquired dentofacial deformities, tooth decay a history of neurological treatment and of orthodontic and/or functional orthopedic treatment, and orofacial myofunctional treatment performed before or during the study.

The 28 children of the posterior crossbite group were treated while participating in all initial stages of the study, but seven of them did not participate in the final exam after palatal expansion and were excluded from the sample. Thus, the crossbite group was reduced to 21 children, 14 of them with unilateral posterior crossbite associated with anterior open bite and 4 with bilateral crossbite. Descriptive data and statistical analysis according to age are presented in Table 1.

**Table 1:** Sex and age of the groups studied.

Groups	N	Sex		Mean age	Standard deviation	*p-*Value
		F	M			
Crossbite	21	10	11	9.2	1.04	0.74
Control	11	6	5	9,3	0,92	

N, number of participants; F, female; M, male.

Student *t*-test for independent samples.

### Otorhinolaryngologic assessment

Evaluation of the upper airways was performed in the Service of Otorhinolaryngology, consisting of anterior rhinoscopy and nasofibroscopy (pediatric flexible fibroscope Pentax FNL – 10RP2, 3.4 mm) for the measurement of adenoid size. Ten of the 21 children in the crossbite group (47.62%) showed 10%–50% obstruction of the cavum, 6 (28.58%) showed 50%–70% obstruction and 5 (23.80%) 70%–100% obstruction. Seven of the 11 control children (63.64%) showed 10%–50% obstruction of the cavum, 2 (18.18%) showed 50%–70% obstruction and 2 (18.18%) 70%–100% obstruction.

### Orthodontic procedures

Complete orthodontic documentation (lateral and occlusal cephalometric radiographies, study models, intraoral photographs and extraoral front and profile photographs) was requested for diagnosis and planning of dental treatment. After the individual study of each orthodontic documentation, a dental mucous-supported orthodontic appliance — modified Haas expander^9^ was prepared. The orthodontic procedures were performed by two dentists, one of them an orthodontics specialist responsible for the installation and adaptation of all appliances. The procedure was finalized by over correction of the uni or bilateral posterior crossbite, taking care for the bite not to cross in a contrary manner. The appearance of a diastema was also noted between the upper central incisors, which was confirmed radiographically with the opening of the median palatal suture in all cases. After this process, which lasted about 15 days, the screw was locked with photopolymerizable resin and the appliance was left in place for containment, without activation, for a period of 180 days for fiber reorganization and bone neoformation in the median palatal suture.^14^

### Videofluoroscopy evaluation

Videofluoroscopy evaluation was performed by the same investigator before the installation of the appliance and 5-months, on average, after its removal. During this time, the stabilization of occlusion was controlled by the orthodontist.

A Philip instrument, model BV Pulsera was used. The images were recorded ona Philips DVD + RW using a recorder coupled to the Medical DVD Recorder, MDVDR – 100 instruments. During the exam, the children sat comfortably on a chair in erect posture wearing a lead apron for radiologic protection. The videofluoroscopic images were capured at a rate of acquisition of 30 frames per second in the lateral and anteroposterior view, with an anterior limit at the lips and a posterior limit at the vertebrae. Mean time of exposure to radiation was 2.0 min for each exam. The images were analyzed using a Vegas Pro.0 image editing software (Sony) that permitted frame by frame analysis and time recording in seconds.

Two Bono® Nestlé cookies were offered to each child (one in a lateral view and the other in an anteroposterior view). Each cookie weighed 11 g and was shaped as a chocolate-flavored mini-pie with Bariogel® 100% barium sulfate (Cristália laboratory, Itapirica, SP, Brazil). The children were instructed to eat the cookies in the usual manner and their natural mastication and swallowing behavior was investigated.

The variables assessed were oral preparatory transit time, oral swallowing transit time, number of cycles and masticatory frequency. For the investigation of preparatory oral transit time and of oral swallowing transit time we considered the definition of the swallowing phases proposed by Logemann.^15^ The author considered the oral preparatory phase as the period of preparation of the food bolus corresponding to mastication, and the oral phase of swallowing as the time of propulsion of the food bolus.

**Figure 1:**
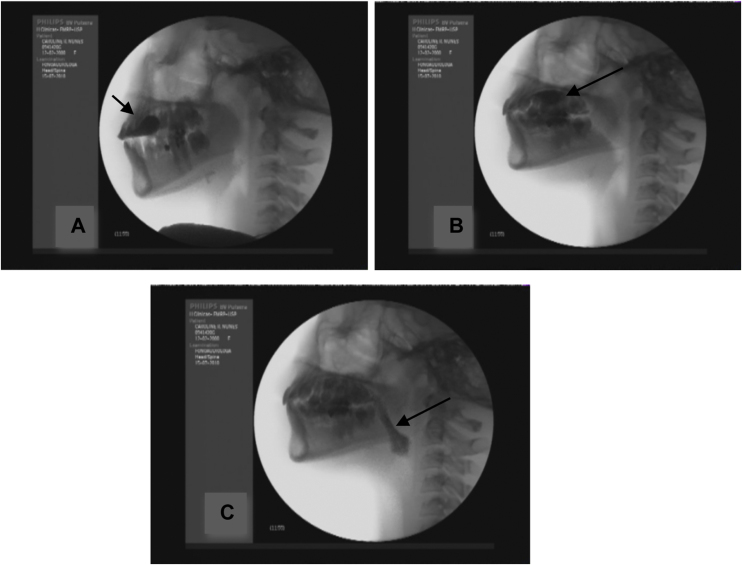
(A) Beginning of the oral preparatory phase; (B) bolus organization; (C) final oral phase.

For the measurement of oral preparatory transit time (seconds) we considered the time from the first movement of bolus capture to bolus organization, and for the measurement of oral transit time we considered the time from the beginning of bolus propulsion to the point when the last portion of the contrasted bolus passes the base of the tongue and crosses the mandible (Fig. 1).

**Figure 2:**
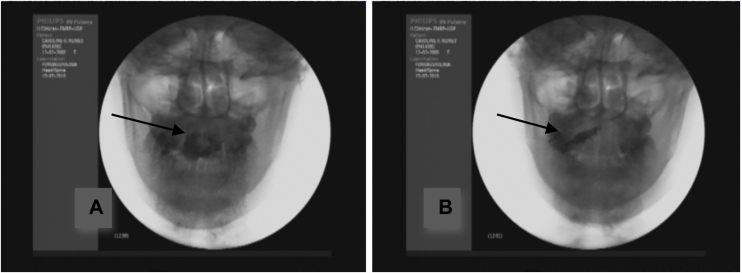
(A) Food cutting; (B) bolus trituration (right side).

Masticatory frequency was calculated as number of masticatory cycles per total time of bolus trituration, in seconds.^16^ Time started to be counted at the beginning of food cutting, ending with the final chewing stroke before the complete organization of the food bolus. The count of number of cycles and the masticatory frequency were considered by analysis in the anteroposterior view (Fig. 2).

### Statistical analysis

The SPSS statistical package, version 17.0, was used for all analyses and the level of significance was set at *p* < 0.05. The Student *t*-test was used for group comparisons regarding all variables studied. The paired *t*-test was used to compare the crossbite group before and after RME and the unpaired *t*-test was used for comparison of the crossbite group and the control group. The *p-*values were adjusted using the false discovery rate method to control the multiple comparisons.^17^

## Results

There was no statistically significant difference (*p* > 0.05) in preparatory oral and oral transit times, number of cycles nor masticatory frequency between the groups studied (Table 2).

**Table 2:** Comparison of the control and crossbite groups before and after orthodontic treatment. Data are reported as mean and standard deviation.

Variables	Control				Crossbite, pre				Crossbite, post			
	N	Mean	SD	P^a^	N	Mean	SD	P^b^	N	Mean	SD	P^c^
PTT (s)	11	7.60	2.24	0.42	21	8.52	2.93	0.15	21	6.98	1.95	0.42
OTT (s)	11	0.84	1.41	0.57	21	0.59	0.27	0.57	21	0.54	0.35	0.57
Cycles	11	9.45	2.33	0.47	21	10.83	3.79	0.47	21	10.21	3.10	0.48
Mast. Freq.	11	1.44	0.28	0.20	21	1.97	1.13	0.20	21	1.61	0.38	0.20

P, probability in the Student *t*-test for independent and dependent samples; PTT, oral preparatory transit time in seconds; OTT, oral transit time in seconds; s, seconds; Mast. Freq.: masticatory frequency (strokes/seconds).

The *p*-values were adjusted using false discovery rate method.

a Comparison of the control group and crossbite group before treatment.

b Comparison of the crossbite group before and after treatment.

c Comparison of the control group and crossbite group after treatment.

## Discussion

Videofluoroscopic evaluation is considered to be the gold standard method for the study of swallowing dynamics and disorders,^18^ permitting a detailed qualitative and quantitative intraoral analysis of the swallowing phases of natural foods.^12^

In the present study, the mastication and swallowing parameters evaluated by videofluoroscopy were similar between patients with posterior crossbite before and after treatment with RME and control subjects. It should be remembered that the duration of the swallowing phases can be influenced by age^19^ and by the consistency of the food tested,^20^ variables that were properly controlled in the present study.

We found no significant difference between groups regarding oral transit time. According to Logemann,^15^ the oral transit time of swallowing occurs within a short period not exceeding 1 or 2 s. These values are compatible with the time observed in the present study, which was 0.65 s on average. These results are similar to those reported by Martín et al.,^21^ who detected no change in swallowing pattern after orthodontic treatment for the correction of posterior crossbite.

We did not detect a significant difference regarding the number of masticatory cycles or masticatory frequency between the groups studied. The mean values obtained for masticatory frequency were 1.97 cycles/s before treatment and 1.61 cycles/s after RME for the children with posterior crossbite and 1.44 cycles/s for the control children with no occlusal changes. We expected to detect a difference between the groups studied since malocclusion may interfere with masticatory performance. Divergent results have been reported in the literature regarding the impact of malocclusion on masticatory performance.^22^, ^23^, ^24^ Some studies have emphasized the influence of some aspects such as quantity of saliva, number of posterior teeth, bite strength and the characteristics of the food itself on the duration of masticatory cycles,^24^, ^25^, ^26^, ^27^ aspects that also impair a direct comparison of our results with those obtaiend in other studies on children in the same age range. Despite a methodological difference compared to the present study, Throckmorton et al.^7^ observed longer masticatory cycles in children aged 7–11 years with posterior crossbite and a reduction of these cycles after RME, reaching the same values as observed in the control group of children without crossbite. Despite the changes, the authors emphasize that the treatment did not change the abnormal form of the masticatory cycle of the patients.

Gambareli et al.^16^ did not detect a difference in masticatory frequency or number of cycles before and after oral rehabilitation in children in the deciduous dentition phase.

In the literature we found studies that use videofluoroscopy mainly in cases of dysphagia. The present study is innovative by using this instrument of investigation to assess the characteristics of mastication and swallowing in children with posterior crossbite before and after orthodontic treatment.

It should be noted that all ethical care and safety of participants during the examination were taken. The time of exposure to radiation was short (average of 2.0 min), with the receipt of a minimum dose (less than 1 Gy of radiation), an amount considered safe by the International Commission on Radiological Protection. However, submission to the videofluoroscopy examination may have inhibited the participation of more subjects in the study.

The small number of participants in the study may have influenced the results, especially in the control group (n = 11). In the crossbite group, seven patients failed to undergo the second assessment after RME, which is considered a loss of 25% of the initial sample (n = 28).

Randomized clinical trials are needed in the future for a better understanding of the relationship between occlusion and orofacial myofunctional condition, an important aspect for professionals who work with the rehabilitation of these patients.

## Conclusion

The applied orthodontic treatment did not influence oral preparatory and oral transit times, nor the number of cycles and masticatory frequency, during biscuit chewing by videofluoroscopy. However, the results of the present study must be interpreted with caution, considering the small sample size.

## Author’s contribution

Tais H. Grechi: Acquisition of data, data interpretation, article writing, final approval.

Carla E. Itikawa: Critical review, final approval.

Fernanda W. M. Gallarreta: Study design, acquisition of data, critical review, final approval.

Wilma Terezinha Anselmo-Lima: Critical review, final approval.

Fabiana C. P. Valera: Critical review, final approval.

Luciana V. V. Trawitzki: Data interpretation, article writing, critical review, final approval.

## Conflicts of interest

The authors declare no conflicts of interest.

## Acknowledgements

This research did not receive any specific funding from funding agencies in the public, commercial, or not-for-profit sectors.
